# A novel coagulation-related lncRNA predicts the prognosis and immune of clear cell renal cell carcinoma

**DOI:** 10.1038/s41598-023-43065-2

**Published:** 2023-09-28

**Authors:** Wensong Wu, Fan Chang, Jianghui Zhang, Shuai Tang, Zhen Lv, Fangmin Chen

**Affiliations:** 1 Department of Urology, The Third Central Clinical College of Tianjin Medical University, Tianjin, China 300170; 2 Department of Urology, The Third Central Hospital of Tianjin, 83 Jintang Road, Tianjin, China 300170; 3 Department of Urology, Nankai University Afnity the Third Central Hospital, Tianjin, China 300170

**Keywords:** Cancer, Computational biology and bioinformatics

## Abstract

Renal cell cancer is associated with the coagulation system. Long non-coding RNA (lncRNA) expression is closely associated with the development of clear cell renal cell carcinoma (ccRCC). The aim of this study was to build a novel lncRNA model to predict the prognosis and immunological state of ccRCC. The transcriptomic data and clinical data of ccRCC were retrieved from TCGA database, subsequently, the lasso regression and lambda spectra were used to filter prognostic lncRNAs. ROC curves and the C-index were used to confirm the predictive effectiveness of this model. We also explored the difference in immune infiltration, immune checkpoints, tumor mutation burden (TMB) and drug sensitivity between the high- and low-risk groups. We created an 8 lncRNA model for predicting the outcome of ccRCC. Multivariate Cox regression analysis showed that age, tumor grade, and risk score are independent prognostic factors for ccRCC patients. ROC curve and C-index revealed the model had a good performance in predicting prognosis of ccRCC. GO and KEGG analysis showed that coagulation related genes were related to immune response. In addition, high risk group had greater TMB level and higher immune checkpoints expression. Sorafenib, Imatinib, Pazopanib, and etoposide had higher half maximal inhibitory concentration (IC_50)_ in the high risk group whereas Sunitinib and Bosutinib had lower IC_50_. This novel coagulation-related long noncoding RNAs model could predict the prognosis of patients with ccRCC, and coagulation-related lncRNA may be connected to the tumor microenvironment and gene mutation of ccRCC.

## Introduction

Clear cell renal cell carcinoma (ccRCC), which accounts for 70% of renal cell carcinoma (RCC) occurrences, is the most prevalent form of RCC, one of the most frequent malignant tumors of the urinary system^1^. The disorder is more prevalent in males, with a male-to-female ratio of around 1.515. More than 140,000 patients died of kidney cancer every year^2^. With mentioned expanding popularity of imaging technologies, more and more persons with ccRCC are being diagnosed every year, thus the treatment is becoming more critical. The medical treatment for ccRCC has progressed, from broad immunological approach to the use of specialist targeted treatments using molecularly targeted medications, and immunotherapy^3^. The molecularly targeted treatments seem to be particularly significant for ccRCC since it is widely recognized for being resistant to chemotherapy and radiation. It is consequently vital to have a good grasp of biological processes and to look into novel, personalized remedies.

The relationship between the coagulation system and malignant tumors was being supported by a growing body of research. Blood would show up in a hypercoagulable condition in a patient who develops a tumor^4^. Additionally, venous thrombosis occur four times more often in tumor patients than in healthy individuals ^5^. Anticoagulants may increase survival time and decrease tumor size in mice tumor models, demonstrating that coagulation factor may be involved in the development of tumors^6^.

Long non-coding RNA (lncRNA) regulates gene expression^7^. More and more studies have shown that lncRNA is involved in tumor progression and can be used as a marker to predict the prognosis of patients^8^.

The association between the immunological microenvironment and the prognostic significance of coagulation-related lncRNAs in ccRCC remain unclear. A novel lncRNA model will be built in this research to predict the prognosis and immunological state of ccRCC.

## Method

### Data extraction

The transcriptomic data and clinical data of ccRCC were retrieved from TCGA database, including 541 tumor samples and 72 normal samples. Then the perl script was used to differentiate between mRNA and lncRNA. In addition, simple nucleotide variation (SNV) data and masked somatic mutation data of ccRCC was downloaded from TCGA database in order to calculate mutational burden. Supplementary data from International Cancer Genome Consortium (ICGC) database was downloaded for validating the effectiveness of the model in ccRCC.

### Identifying the differentially expressed coagulation-related lncRNAs

The Molecular Signature Database (MsigDB) provided a total of 139 coagulation-related genes (CRGs)^9^, and Pearson correlation analysis was used to determine the relationship between CRGs and coagulation-related lncRNAs. Those lncRNAs were thought to be coagulation-related lncRNA when Pearson’s correlation coefficient was higher than 0.5 and p value lower than 0.001.

### Screening of coagulation-related lncRNAs related to prognosis

Coagulation-related lncRNAs associated with the prognosis of patients with renal cell carcinoma were identified using univariate cox regression analysis by combining LncRNA expression data with survival data, then used the “pheatmap” package to visualize^10^.

### Consensus clustering

The coagulation-related lncRNAs related to prognosis were selected for unsupervised clustering by using “ConsensusClusterPlus” package. The proposed cluster number varied from two to nine and 1000 replications were conducted to obtain the most reliable classifcation.

### Construction of coagulation-related lncRNAs prognostic model

The ccRCC data were split into two groups at random: the training group was used to build the risk model, while the validation group was used to evaluate the model. The correlation coefficient of each lncRNA is represented by inCoef(i), while the expression level of a lncRNA is represented by Expr(i). Subsequently, the lasso regression and lambda spectra were used to filter lncRNAs, the risk score = **∑**inCoef(i) × Expr(i). Following model creation, the overall survival (OS) of high and low risk subgroups in the test group and the verification group were compared. We then employed the ROC curve and consistency index to evaluate the model's correctness.

### GO and KEGG and principal components analysis

Gene expression levels between the low-risk and high-risk group was compared using a differential analysis. GO and KEGG analysis were used to analyze the pathways enriched by differential genes. The “scatterplot3d” was used to visualize the expression of coagulation-related lncRNAs in ccRCC.

### Immune analysis

CIBERSORT was used to assess the level of immune cell infiltration in ccRCC, and spearman analysis to determine the correlation between risk score and immune cell infiltration level. The “ggpubr” package was used to compared immune checkpoint activity between low-risk and high-risk group.

### Tumor mutation analysis and drug sensitivity analysis

The “Maftools” package was used to count and visualize the nonsynonymous point mutations in each sample. Additionally, the survival rates of patients with various tumor mutation burden (TMB) and risk score were compared, as well as the tumor mutation loads of the high risk and low risk groups. The “pRRophetic” package was used to compare the half maximal inhibitory concentration (IC_50_) of different drugs in the high-risk and low-risk groups.

## Results

### Coagulation-related lncRNAs with prognostic significance

**Figure 1:**
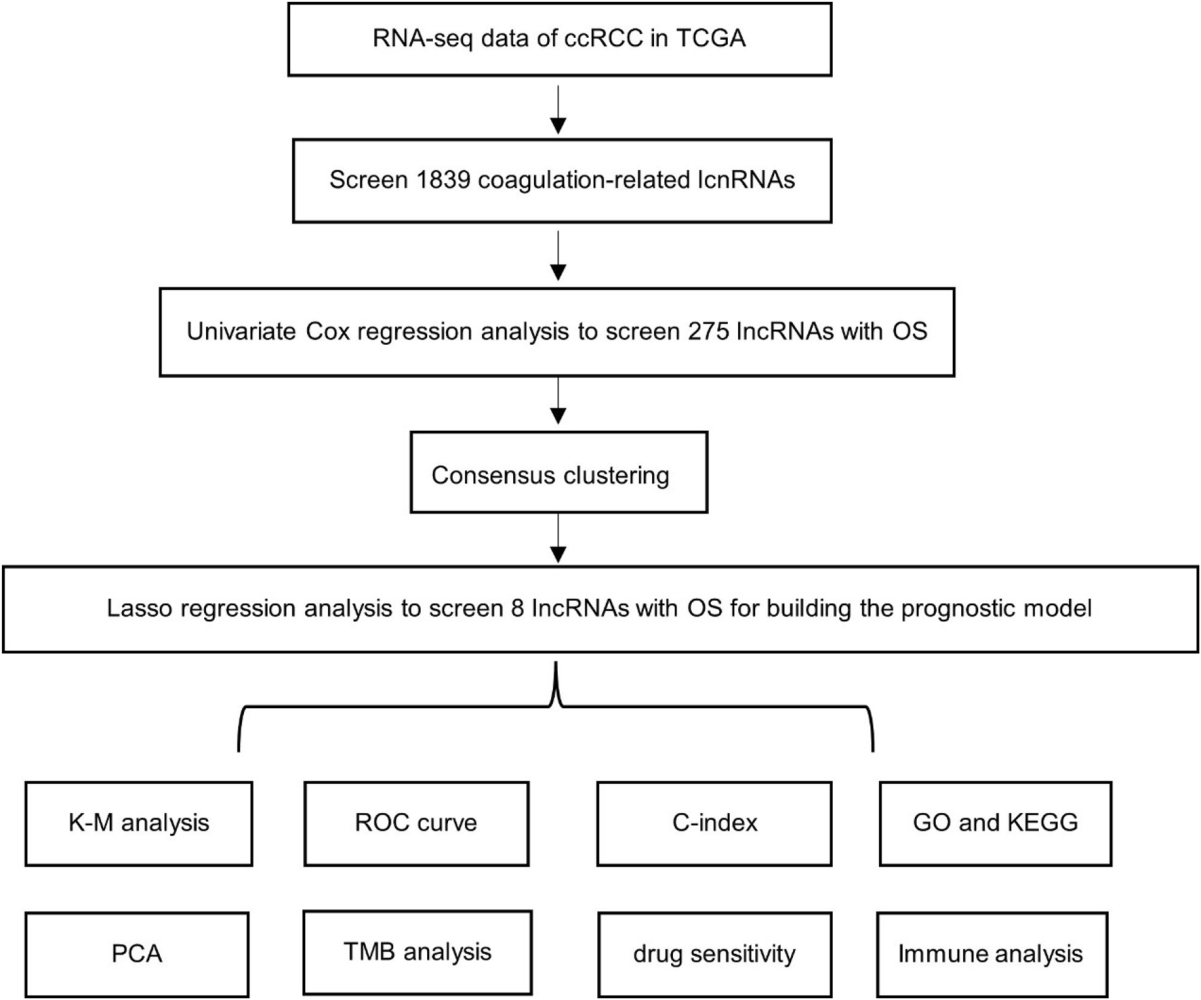
The study workflow.

**Figure 2:**
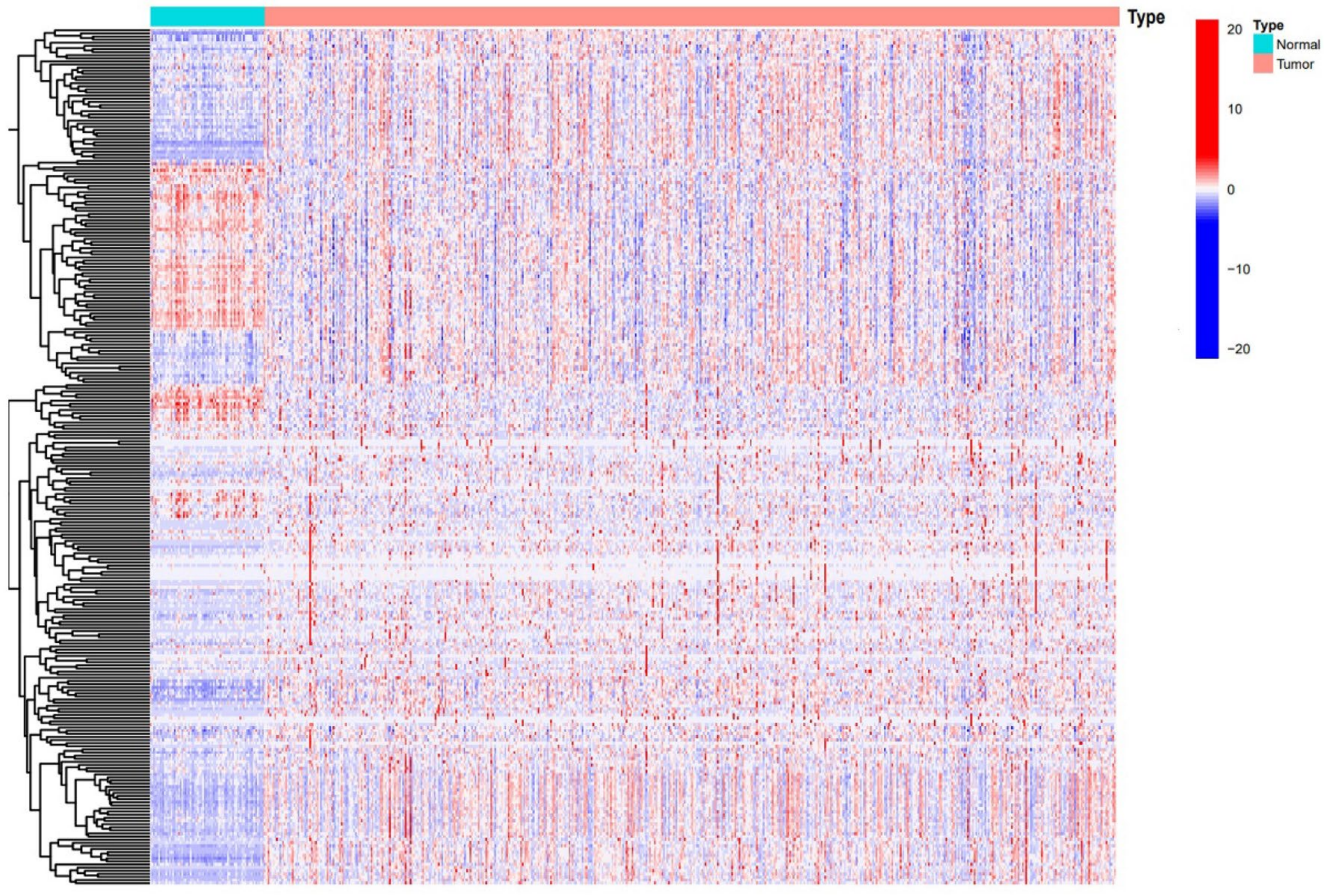
The heatmap of prognostic coagulation-related lncRNA model.

The study flowchart is depicted in Fig. 1. By correlating lncRNAs with genes linked to coagulation (|Pearson R|> 0.5 and p < 0.001), 1839 coagulation-related lncRNAs were found, and 275 coagulation-related lncRNAs associated to the prognosis of ccRCC were screened using the univariate cox analysis. The heatmap demonstrated that the expression of coagulation-related lncRNAs associated with prognosis differs significantly between tumor tissues and healthy tissues (Fig. 2).

### Novel two molecular subtypes of ccRCC

**Figure 3:**
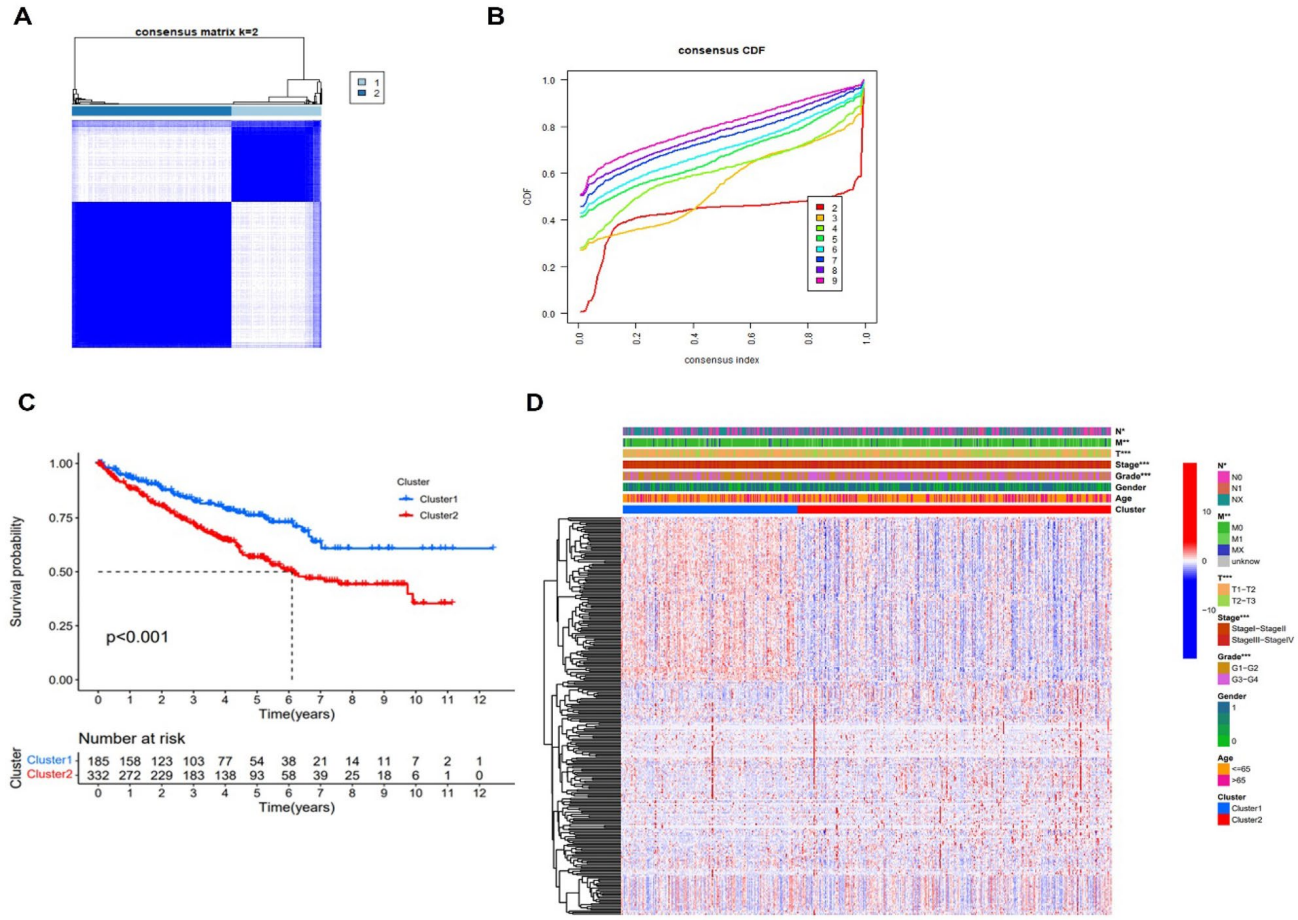
Classifcation of cluster. (**A**) Consensus clustering matrix. (**B**) CDF curve. (**C**) K–M curve of different subtypes. (**D**) The heatmap of clinical characteristics. *P < 0.05, **P < 0.01, *P < 0.001.

According to the differential expression of 275 coagulation related lncRNAs associated to the prognosis, the complete sample was partitioned into distinct clusters, and the consensus CDF curve indicated that k = 2 was the best partition. In the TCGA database, ccRCC patients could be typically classified into two molecular subgroups, Cluster1 (C1) and Cluster2 (C2) (Fig. 3A,B), and K-M curve showed that C2 having a considerably poorer overall survival rate than C1 (Fig. 3C). Further research has shown that the C2 subtype was more linked to worse clinical and pathological characteristics than the C1 subtype, according (Fig. 3D).

### Construction of coagulation-related lncRNAs model

**Figure 4:**
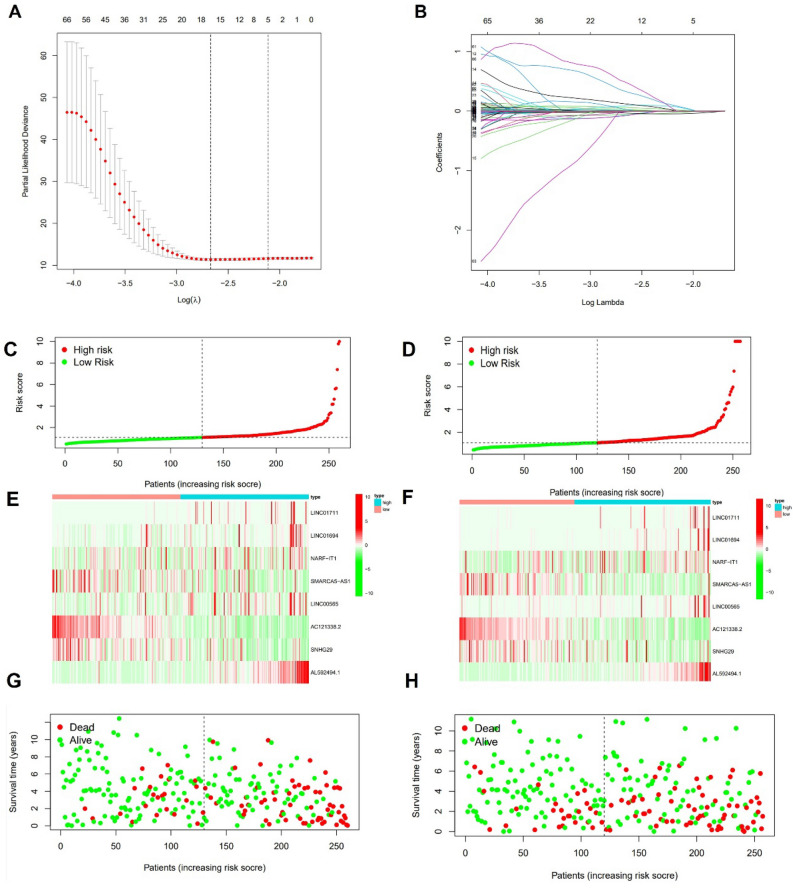
Construction of coagulation-related lncRNA model. (**A**) Lasso regression of prognostic coagulation-related lncRNA model. (**B**) Cross-validation for tuning parameter selection in LASSO regression. (**C**,**D**) The risk score plot. (**E**,**F**) lncRNA expression in train set and validation set. (**G**,**H**) Survival status plot.

We created a model for predicting the outcome of ccRCC using lasso regression analysis based on the 275 coagulation-related lncRNAs associated prognosis that were described before (Fig. 4A,B).

**Figure 5:**
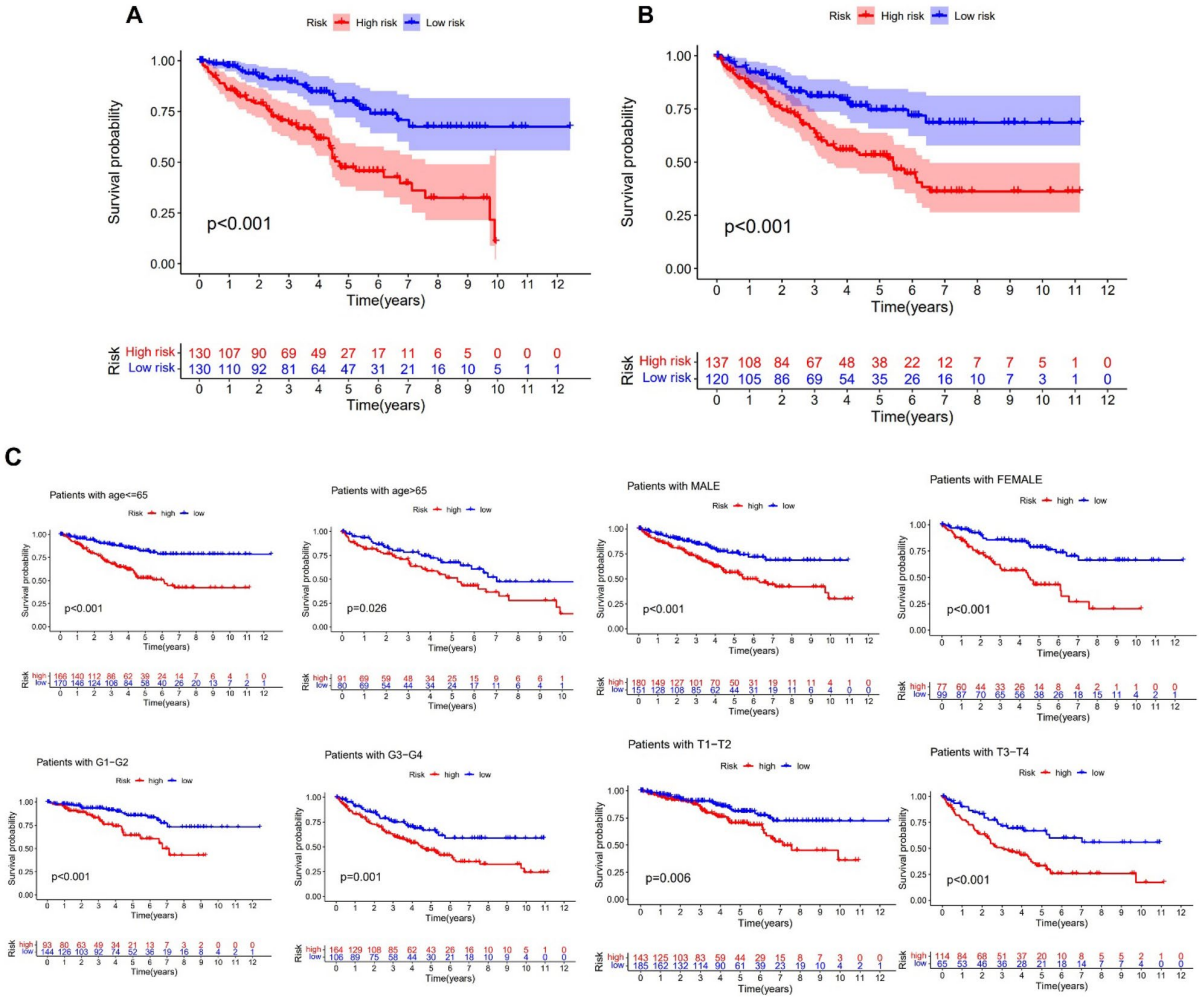
K–M survival curve of OS. (**A**) Train set. (**B**) Validation set. (**C**) Different clinical characteristics.

Following the selection of 8 lncRNA as model genes, the risk score was determined in the manner described below for ccRCC patients: LINC01711 × (0.0037830) + LINC01694 × (0.2212711) + NARF-IT1 × (0.0051376) + SMARCA5-AS1 × (− 0.0281499) + LINC00565 × (0.0569755) + AC121338.2 × (− 0.0822645) + SNHG29 × (− 0.0001535) + AL592494.1 × (0.7739774). Based on the median risk score, we divided the sample into high- and low-risk groups. The Fig. 4C–H demonstrated the training and validation groups' risk core, survival status, and gene expression. According to the training and validation groups' survival outcomes, patients in the high-risk group had a considerably lower OS than those in the low-risk group (Fig. 5A,B). Additionally, individuals in the high-risk category had a worse OS similarly in various features (Fig. 5C). In order to validate the effectiveness of the coagulation-related lncRNA model, we compared the differential survival rates between the high-risk and low-risk groups using external data. The results demonstrated that patients in the high-risk group exhibited lower OS (Supplementary Figure).

### Evaluation of the predictive power of the model

**Figure 6:**
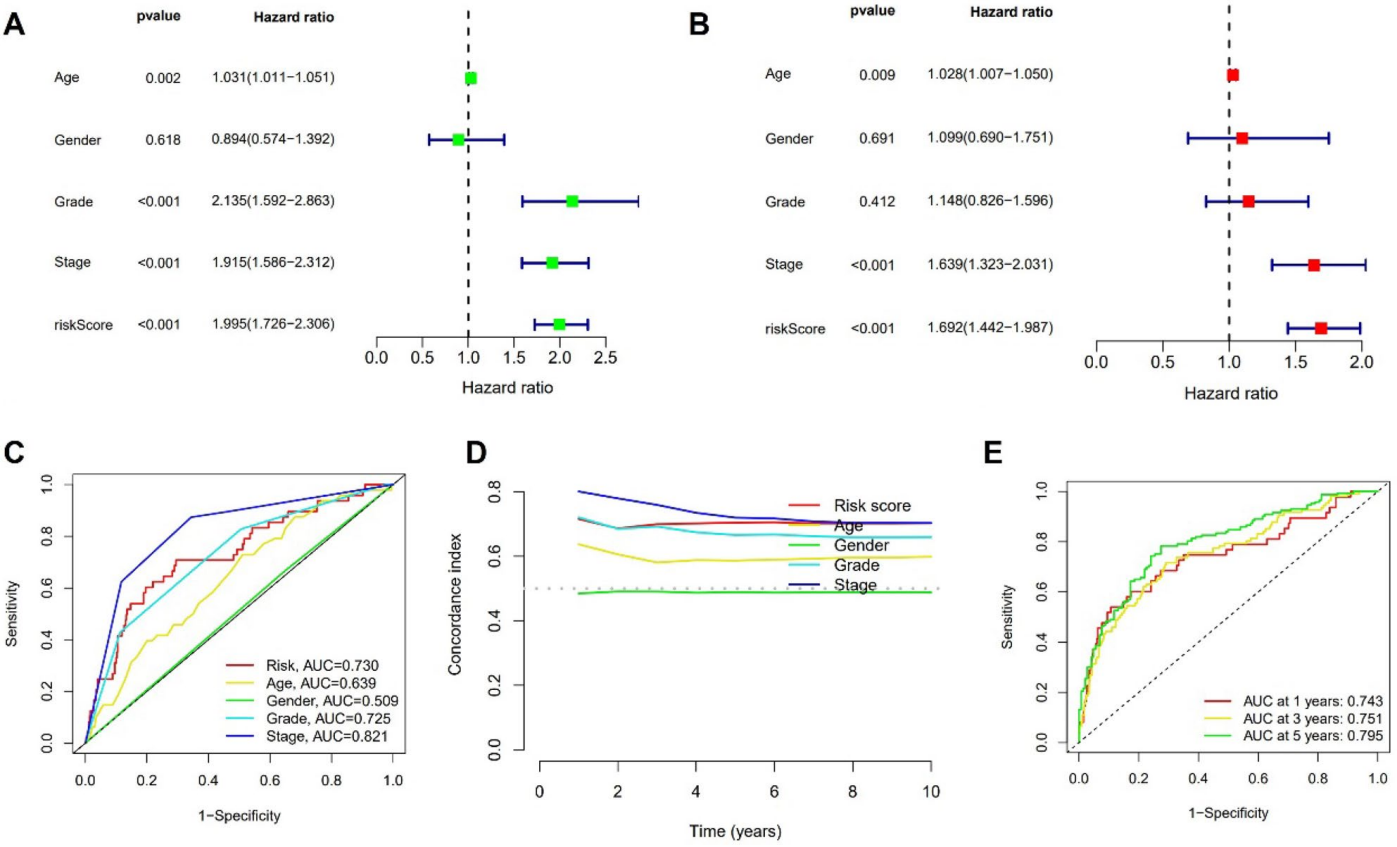
Evaluation of the predictive power of the model. (**A**,**B**) Univariate and Multivariate Cox regression analysis. (**C**) ROC curve taking consideration of risk score and clinical characteristics. (**D**) C-index curve. (**E**) ROC curve of 1-year, 3- year, and 5-year overall survival.

Univariate and multivariate Cox regression analysis showed that age, tumor grade, and risk score are independent prognostic factors for ccRCC patients (Fig. 6A,B). The predictive power of the model was second only to tumor stage, with the AUC of risk score being greater than that of age, sex, and tumor grade (Fig. 6C). The C index produced identical findings (Fig. 6D). The model could assess the prognosis of patients with ccRCC, as shown by the AUC of ROC curves for predicting 1-year, 3-year, and 5-year overall survival, which were 0.743, 0.751, and 0.795, respectively (Fig. 6E).

### Functional enrichments analysis and PCA

**Figure 7:**
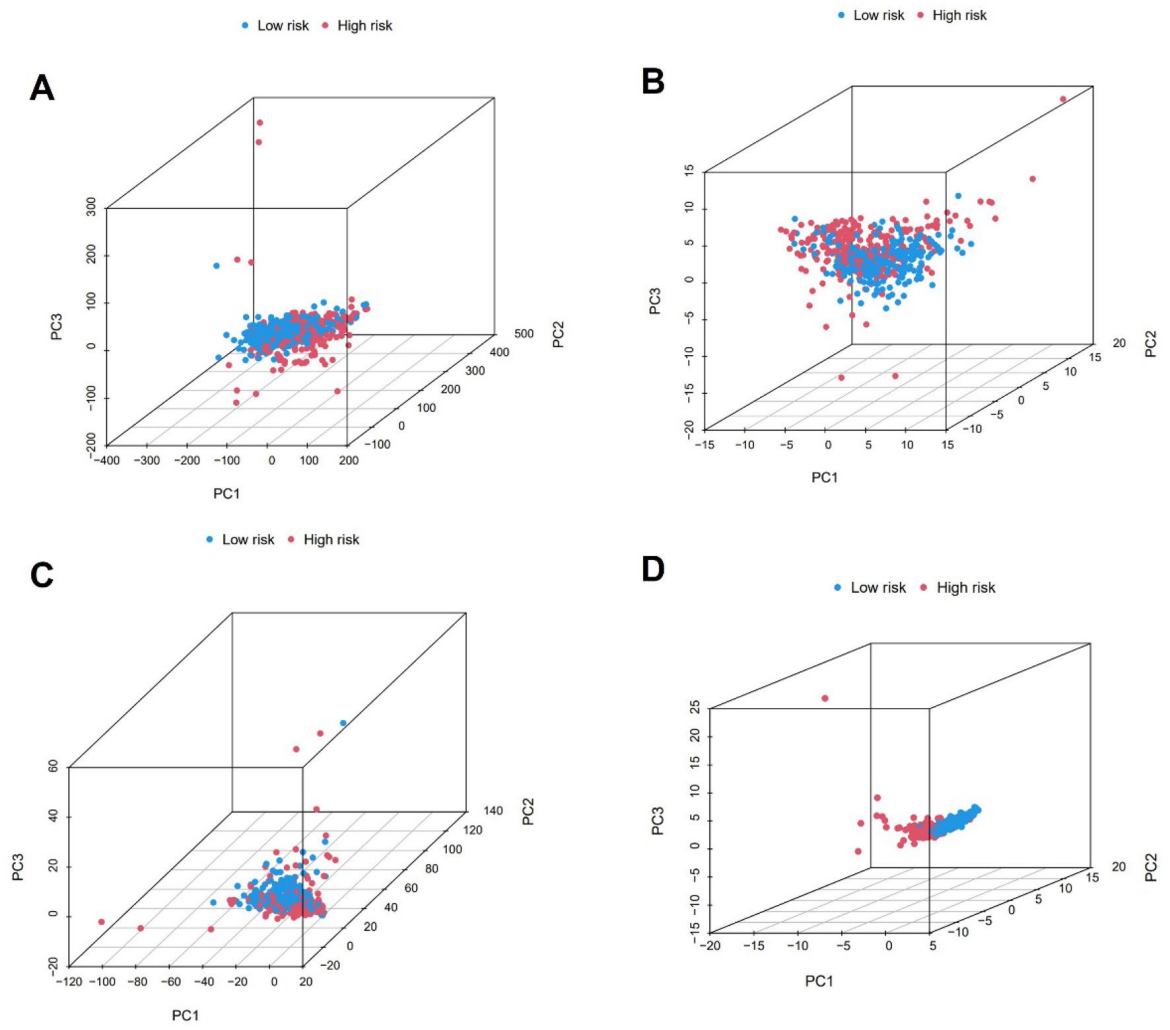
PCA analysis of lncRNA. (**A**) All gene module. (**B**) Coagulation-related gene module. (**C**) Coagulation-related lncRNAs module. (**D**) Coagulation-related lncRNAs prognostic module.

**Figure 8:**
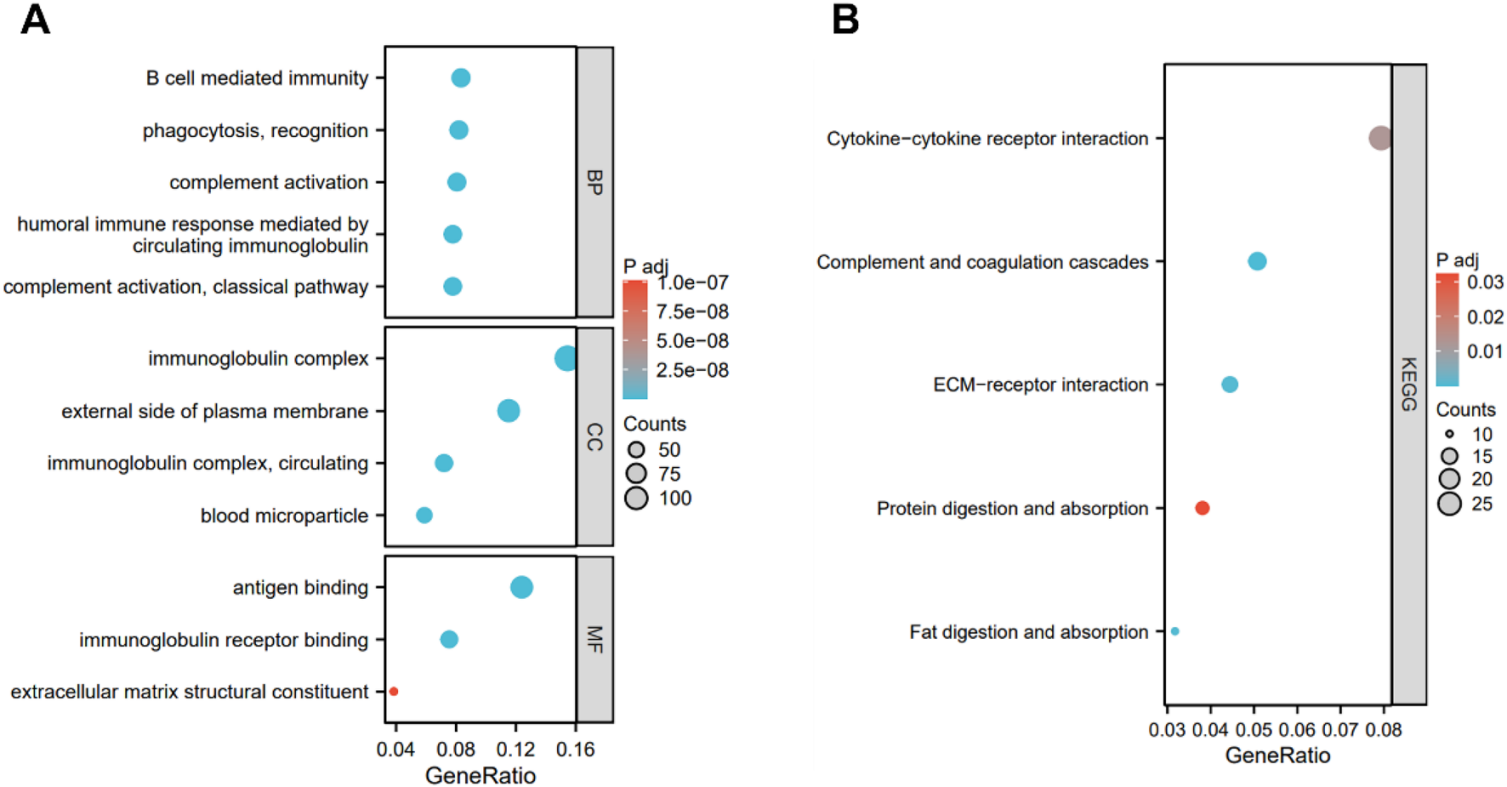
Functional enrichment analysis. (**A**) GO analysis. (**B**) KEGG analysis.

In comparison to other modules, results demonstrated that the coagulation-related lncRNA prognostic module could clearly discriminate between high risk group and low risk group (Fig. 7A–D). We performed GO and KEGG pathway analysis to comprehend the activities of genes that were differently expressed across high-risk and low-risk groups. GO analysis results showed that these differential genes are linked to B cell mediated immunity, complement activation, antigen binding, humoral immune response mediated by circulating immunoglobulin and extracellular matrix structural constituent (Fig. 8A). And KEGG analysis results demonstrated that cytokine–cytokine receptor interaction, complement and coagulation cascades and ECM–receptor interaction were enriched in differential genes (Fig. 8B). These suggested that coagulation related genes are related to immune response and may be involved in the formation of tumor microenvironment.

### TMB

**Figure 9:**
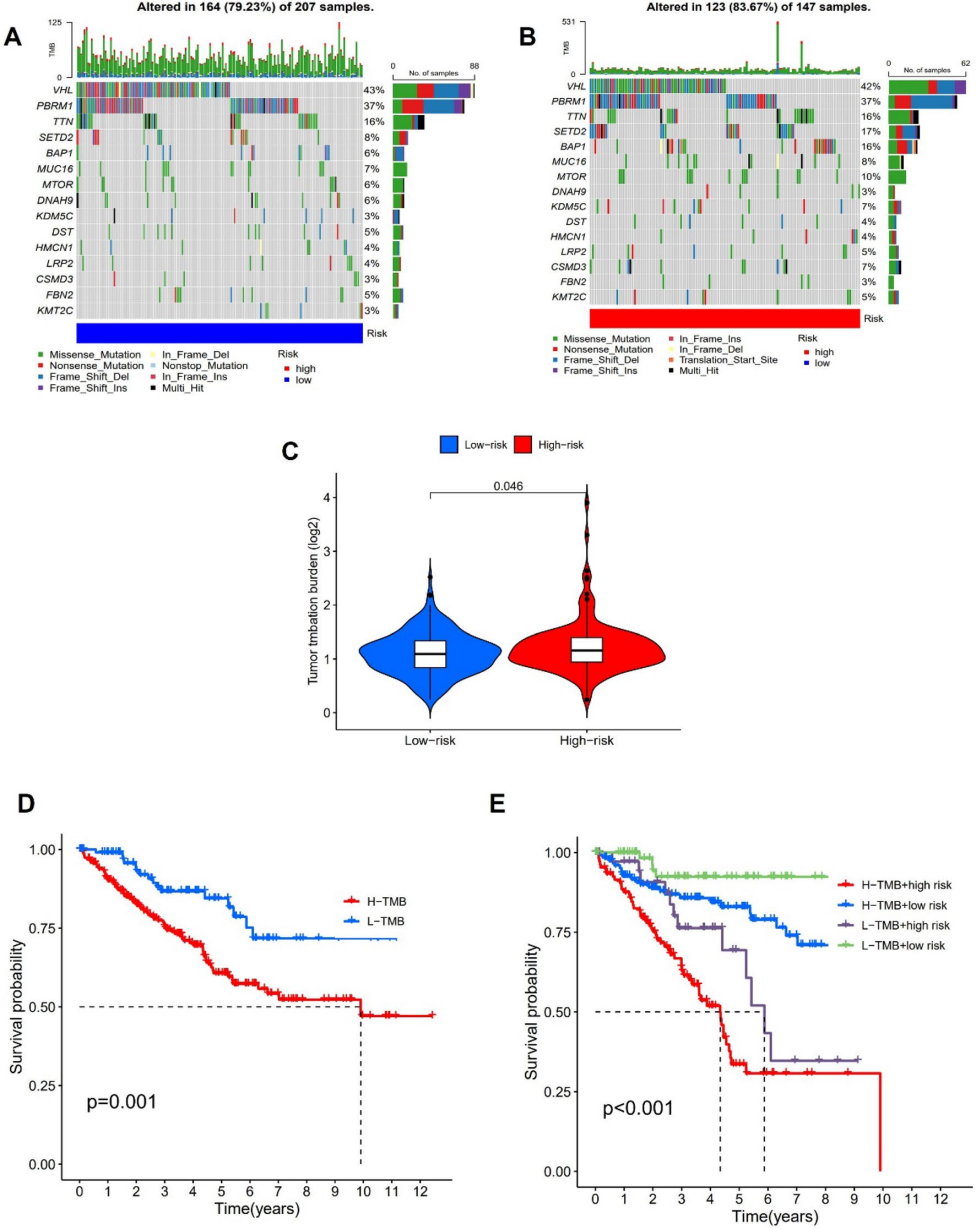
TMB analysis. (**A**,**B**) The waterfall plot showed the somatic mutation rate between high risk group and low risk group. (**C**) Difference in TMB between high risk group and low risk group. (**D**,**E**) K–M curve in different groups of ccRCC patients.

The difference in TMB between the high-risk group and the low-risk group were also examined. We discovered that the high risk group had greater SETD2 and BAP1 mutation rates than the low risk group (Fig. 9A,B). The renal cell carcinoma patients with high TMB level had lower OS (Fig. 9C) and high risk group had greater TMB level in the TCGA database (Fig. 9D). A further indication that coagulation-related lncRNA is connected to prognosis is the fact that patients with high risk and high TMB had the lowest survival rates (Fig. 9E).

### Immune infiltration, immune checkpoints and drug sensitivity

**Figure 10:**
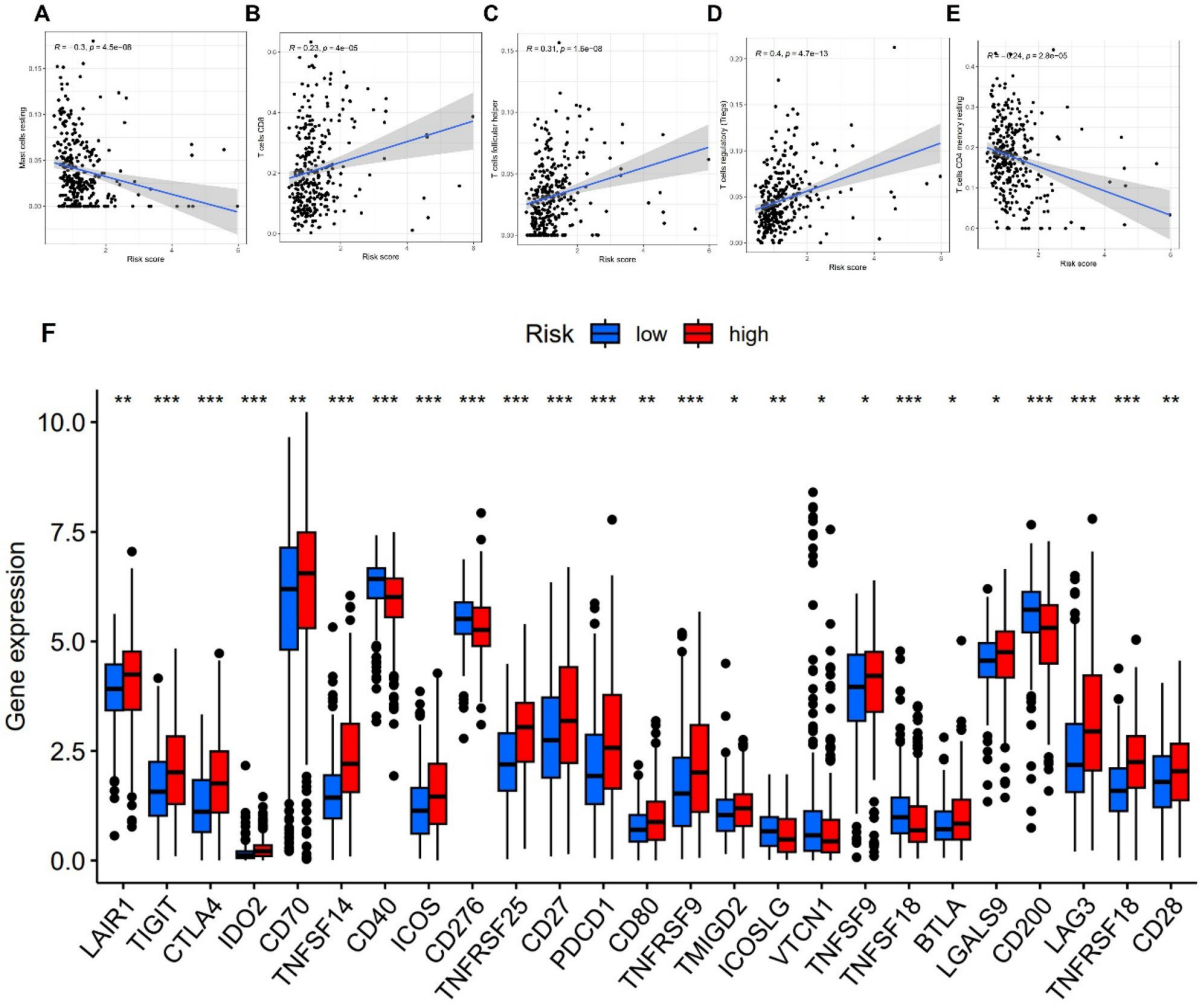
Immune analysis. (**A**–**E**) The relationship between different immune cell infiltration and risk score. (**F**) Difference of immune checkpoint expression between high risk group and low risk group.

**Figure 11:**
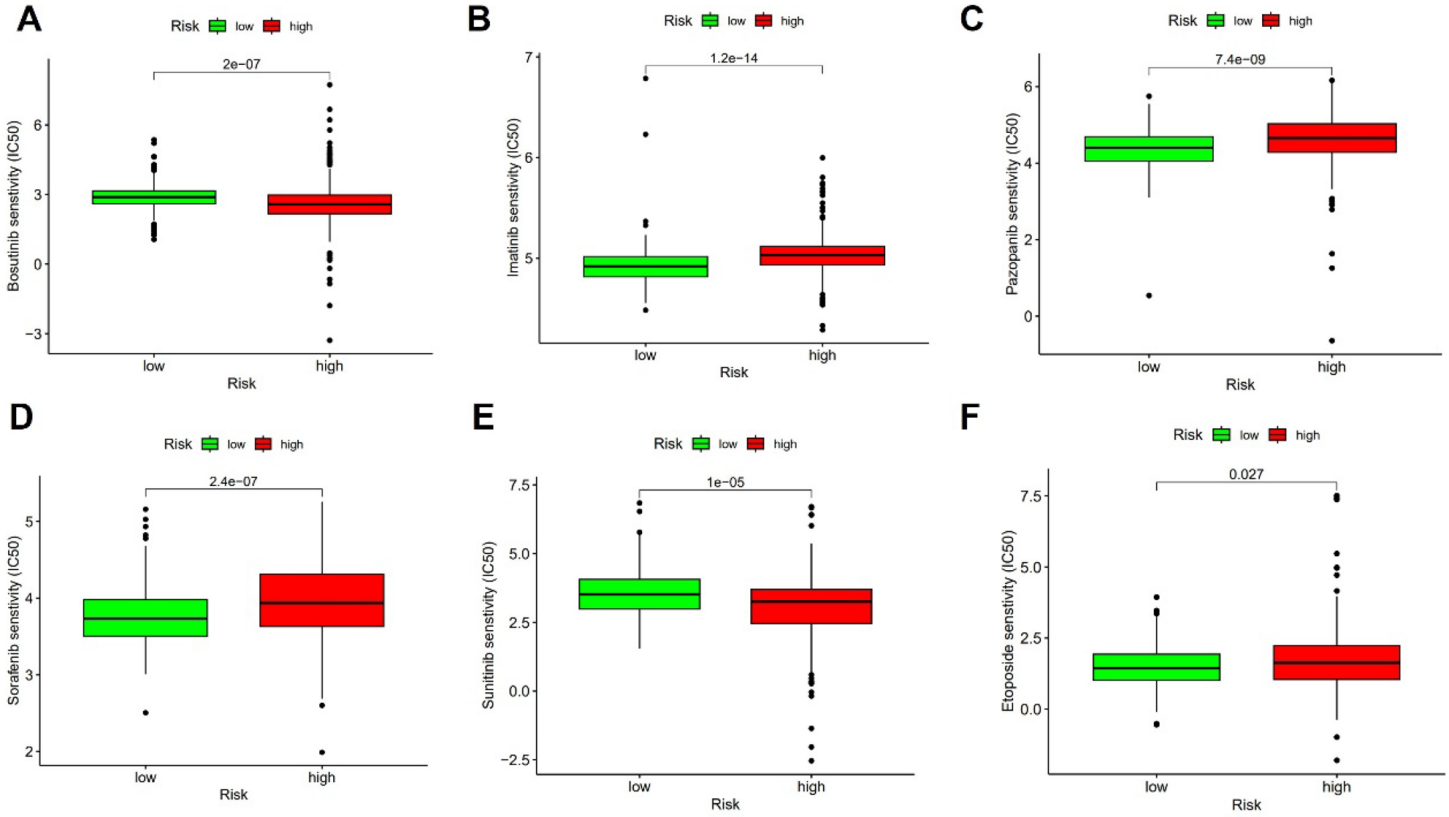
Drug sensitivity analysis. A comparison of the IC50 for several chemotherapy agents in high and low risk groups (**A**–**F**).

The risk score was connected to several levels of immune cell infiltration, including mast cells, regulatory T cells, follicular helper T cells, CD8+ T cells, and CD4+ T cells (Fig. 10A–E). The majority of immune checkpoints, including PDCD1, CTLA4, LAG3, TIGIT, CD27, LAGAS9 were found to be strongly expressed in the high risk group when the expression of immune checkpoints between the high risk group and the low risk group was compared (Fig. 10F). Sorafenib, Imatinib, Pazopanib, and etoposide had higher IC_50_ in the high risk group whereas Sunitinib and Bosutinib had lower IC_50_ (Fig. 11A–F).

## Discussion

Renal cell cancer is associated with the coagulation system. According to studies, tissue factor is independent risk factors for specific mortality in patients with renal cell carcinoma^11^. The increase of plasma coagulation markers fibrinogen, fibrin monomer and D-Dimer is related to the decrease of OS^12,13^. The tumor angiogenesis depends on the coagulation system, and connected to a number of anti-angiogenic medications. The coagulation system is the key to the tumor angiogenesis, and it is also related to a variety of anti-angiogenic drugs. Several targeted treatments for renal cell carcinoma, including Sunitinib, have an anti-angiogenic impact. Drug effectiveness may be increased or drug resistance decreased by understanding the mechanism of the blood coagulation system in renal cell cancer.

For a variety of cancers, lncRNA may be employed as a diagnostic and prognostic marker. In this work, coagulation-related lncRNAs and the prognosis of ccRCC are associated by bioinformatics methods. We first constructed a prognostic model of 8 coagulation-related lncRNAs by lasso regression. Further analysis revealed the model had a good performance in predicting prognosis of ccRCC. GO and KEGG analysis were performed to examine the biological role of coagulation-related lncRNA. The results indicated that coagulation-related lncRNAs was connected to several immune-related pathways, indicating that they may be connected to the immunological microenvironment of renal cell cancer. The tumor immune microenvironment (TIME) refers to the infiltration of numerous immune cells and immune regulatory factors within the context of the tumor microenvironment^14^. The interplay between specific human immunity and tumor immune escape contributes to the formation of the tumor microenvironment. There exist substantial variations in the extent of immune cell infiltration across diverse tumor types^15^. Disparities in immune cell infiltration levels and the expression of immune regulatory factors could potentially contribute to tumor progression^15^. For instance, previous research has indicated lower expression of Th1 and Th17-related markers in tumor tissues^16^. Various factors, including tumor genotype, TMB, and humoral factors, can influence the formation of the tumor microenvironment. Moreover, gaining a deeper understanding of the tumor immune microenvironment's relevance to tumor treatment suggests that immune microenvironment modulators could potentially enhance the therapeutic efficacy in patients unresponsive to ICIs^17^. To further support this theory, we explored the relationship between risk score and immune cells in order. We found that risk score was positively correlated with regulatory T cells. By interacting with other immune cells and producing immune components, regulatory T cells have the potential to significantly contribute to immunological tolerance, indicating that coagulation-related lncRNAs may be connected to tumor immunosuppression. Further research revealed that the high risk group had increased immune checkpoint expression, including PDCD1 and CTLA4The reason the body's anti-tumor response was reduced and not boosted by the increased CD8+ T cell and NK cell populations surrounding the tumor is because it's probable that an immunosuppressive mechanism enabled ccRCC to withstand the fatal effects of NK cells and CD8+ T cells. Immunotherapy, which includes boosting a patient's immune system to assist immune cells in locating and eliminating cancer cells rather than the tumor itself, is now the cornerstone of contemporary cancer treatment^18^. In order to maintain immune homeostasis, immunological checkpoint molecules are essential because they inhibit immune overactivation. ccRCC have an overexpression of immunological checkpoint molecules, which is an important immune evasion strategy^19^. Cytotoxic T lymphocyte antigen-4 (CTLA-4) and Programmed cell death 1 (PD-1) are the two most important immune checkpoint molecules used to avoid immune system detection^20^. Renal cell carcinoma can be effectively treated with immune checkpoint inhibitors, such as the PD-1 inhibitor nivolumab, which is utilized as a second-line therapy for advanced renal cell carcinoma^21^. Despite the initial clinical response, long-term immunotherapy often results in drug-resistant cancers, which presents a significant treatment hurdle^22^. Therefore, awareness of the alleged immunosuppressive mechanisms must be acquired for the purpose for patients with ccRCC to have a decent prognosis. In summary, these findings imply that coagulation-related lncRNAs could someday be a target for ccRCC immunotherapy. Through TMB analysis, we discovered that the high-risk group had higher SETD2 and BAP1 mutation rates. Renal cell carcinoma has a significant mutation rate for the tumor suppressor gene SETD2^23^. The SETD2 mutation has a positive correlation with metastasis, according to genomic research^23^. BAP1 is a multifunction suppressor of cancer that influences the immune system, cell cycle control, DNA damage response via its connection with BRCA1, chromatin remodeling, and DNA damage response^24^. A variety of aggressive malignancies, most notably uveal melanoma, malignant mesothelioma, and renal cell carcinoma, are often caused by mutations in the BAP1 gene^25^. The TMB is associated with the therapeutic effects of tumor immune checkpoint inhibitors (ICIs) and can serve as one of the indicators of ICIs treatment efficacy^26^. A higher TMB leads to the generation of more antigens, facilitating specific recognition by T cells, which could potentially result in better treatment outcomes^27^. Moreover, there exists considerable variation in tumor mutation burdens across different tumors. High TMB tumors cannot predict the efficacy of ICIs treatment for all types of cancers^28^. The utilization of TMB as a prospective biomarker for assessing tumor treatment efficacy encounters ongoing challenges, highlighting the imperative for additional research endeavors to authenticate its validity^29,30^. In our study, Sorafenib, Imatinib, Pazopanib, and etoposide had higher IC_50_ in the high-risk group whereas Sunitinib and Bosutinib had lower IC_50_. The Sorafenib was the first antiangiogenic multikinase inhibitor for RCC to be approved by the FDA^31^. Pazopanib is commonly used in patients with advanced ccRCC, and its therapeutic effect was noninferior to sunitinib^32^. These findings may contribute to a more precise treatment selection process based on the genetic features of coagulation-related lncRNA in various patient malignancies. Future pharmacological treatment plans for people with kidney cancer may be improved as a result of our study.

Our research has several of limitations. First of all, the fact that this research only used data from one database might skew the findings. Second, the degree of coagulation-related lncRNA expression in ccRCC was not independently verified. Finally, the list of genes we listed may not be exhaustive.

In conclusion, this novel coagulation-related long noncoding RNAs model could predict the prognosis of patients with ccRCC, and coagulation-related lncRNA may be connected to the tumor microenvironment and gene mutation of ccRCC. To confirm these results, more research is still necessary.

### Supplementary Information

Supplementary Figure 1.

## Data Availability

All relevant data during this study are within the paper.
