# Supplementary material for: A novel coagulation-related lncRNA predicts the prognosis and immune of clear cell renal cell carcinoma
Source: Sci Rep. 2023 Sep 28;13:16302. doi: 10.1038/s41598-023-43065-2 (PMC10539335; doi:10.1038/s41598-023-43065-2)
Supplement: Supplementary file 1 — Supplementary Figure 1. [file 41598_2023_43065_MOESM1_ESM.docx]

**Supplementary Figure**


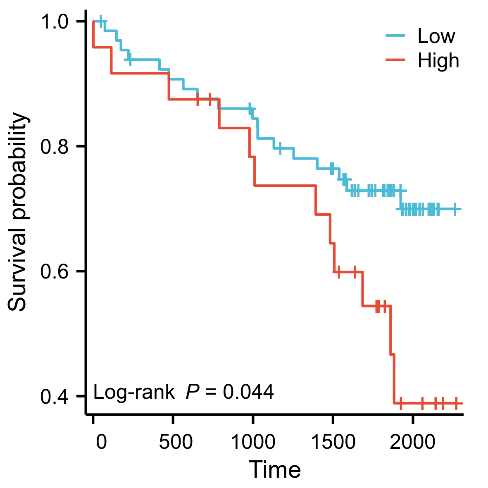


**Supplementary Figure 1.** Kaplan-Meier curves depicting the high and low-risk groups of renal carcinoma patients from the ICGC dataset
